# Local government funding and life expectancy in England: a longitudinal ecological study

**DOI:** 10.1016/S2468-2667(21)00110-9

**Published:** 2021-07-12

**Authors:** Alexandros Alexiou, Katie Fahy, Kate Mason, Davara Bennett, Heather Brown, Clare Bambra, David Taylor-Robinson, Benjamin Barr

**Affiliations:** a Department of Public Health, Policy, and Systems, University of Liverpool, Liverpool, UK; b Population Health Sciences Institute, Newcastle University, Newcastle upon Tyne, UK

## Abstract

**Background:**

Since 2010, large reductions in funding for local government services have been introduced in England. These reductions in funding have potentially led to reduced provision of health-promoting public services. We aimed to investigate whether areas that showed a greater decline in funding also had more adverse trends in life expectancy and premature mortality.

**Methods:**

In this longitudinal ecological study, we linked annual data from the Ministry of Housing, Communities, and Local Government on local government revenue expenditure and financing to 147 upper-tier local authorities in England between 2013 and 2017 with data from Public Health England, on male and female life expectancy at birth, male and female life expectancy at age 65 years, and premature (younger than 75 years) all-cause mortality rate for male and female individuals. Local authorities were excluded if their populations were too small or if changes in boundaries meant consistent data were not available. Using multivariable fixed-effects panel regression models, and controlling for local socioeconomic conditions, we estimated whether changes in local funding from 2013 were associated with changes in life expectancy and premature mortality. We included a set of alternative model specifications to test the robustness of our findings.

**Findings:**

Between 2013 and 2017, mean per-capita central funding to local governments decreased by 33% or £168 per person (range –£385 to £1). Each £100 reduction in annual per person funding was associated over the study period 2013–17 with an average decrease in life expectancy at birth of 1·3 months (95% CI 0·7–1·9) for male individuals and 1·2 months (0·7–1·7) for female individuals; for life expectancy at age 65 years, the results show a decrease of 0·8 months (0·3–1·3) for male individuals and 1·1 months (0·7–1·5) for female individuals. Funding reductions were greater in more deprived areas and these areas had the worst changes in life expectancy. We estimated that cuts in funding were associated with an increase in the gap in life expectancy between the most and least deprived quintiles by 3% for men and 4% for women. Overall reductions in funding during this period were associated with an additional 9600 deaths in people younger than 75 years in England (3800–15 400), an increase of 1·25%.

**Interpretation:**

Our findings indicate that cuts in funding for local government might in part explain adverse trends in life expectancy. Given that more deprived areas showed greater reductions in funding, our analysis suggests that inequalities have widened. Since the pandemic, strategies to address these adverse trends in life expectancy and reduce health inequalities could prioritise reinvestment in funding for local government services, particularly within the most deprived areas of England.

**Funding:**

National Institute for Health Research (NIHR) School for Public Health Research, NIHR Applied Research Collaboration North East and North Cumbria, NIHR Applied Research Collaboration North West Coast and Medical Research Council.

## Introduction

Life expectancy in England has stalled. Although similar trends have been observed in many high-income countries since 2011, the situation in England is among the worst.^1^ These adverse trends in life expectancy have disproportionately affected the most deprived areas, reversing improvements in inequalities accrued over the previous decade.^2^

The reasons for this plateauing remain unclear; that the population has reached its natural biological limits is unlikely, given that life expectancy in other countries is higher and rising.^3^ Most reviews have pointed to multiple factors,^4^, ^5^ including the timing of the smoking epidemic^1^ or cold weather and higher prevalence of influenza.^5^ These factors however do not explain the change in trend from 2011,^4^ or its persistence over several years. A growing number of studies have associated stalling life expectancy with reduced funding for public services following the introduction of austerity measures in England in 2010.^6^, ^7^, ^8^, ^9^ These studies have largely focused on health and social care expenditure and have been based on relatively simple analyses comparing national trends in spending and national trends in mortality, providing weak causal evidence. Studies of the relationship between mortality and public expenditure also struggle with distinguishing between the effects of expenditure on health outcomes and reverse causality, whereby increased expenditure is driven by increases in needs that are determinants of increased mortality.^10^, ^11^

**Research in context**

**Evidence before this study**

We searched PubMed and Google Scholar from inception to March 19, 2021, the date we did our search, for articles in English assessing the effects of local government spending on life expectancy or mortality using the following terms: “local government” or “local services”; “expenditure”, “spending”, or “austerity”; and “life expectancy” or “mortality”. The majority of relevant studies relate to the USA and the UK context, with a growing number of studies in the UK pointing to the association between declining life expectancy and reduced funding for public services after the imposition of austerity measures in 2010. These observational studies have largely focused on health service, public health, and social care spending and have been based on relatively simple analyses of national trends, providing weak causal evidence. To our knowledge, there have been no previous studies in the UK investigating the effects of reduced local government funding on life expectancy and premature mortality.

**Added value of this study**

We investigated whether areas in England that showed a greater decline in allocated local funding between 2013 and 2017, also had greater declines in life expectancy. During this period, we estimated that each £100 reduction in funding per person was associated with a decrease in life expectancy at birth of 1·3 months (95% CI 0·7–1·9) for men and 1·2 months (0·7–1·7) for women. In total, we estimate that reductions in funding during this period were associated with an additional 9600 deaths for people younger than 75 years in England (3800–15 400). Reductions in funding were greater in more deprived areas. We estimate that cuts in funding might have increased the gap in life expectancy between the most and least deprived areas by 3% for men and 4% for women.

**Implications of all the available evidence**

Reductions in funding for local government in England, along with policy changes on how funding is distributed among areas might have adversely affected life expectancy. Local government in England, as in many countries, provides a wide range of services that have an effect on health, including housing, social care, cultural, planning, and environmental services. Our study suggests that reduced funding for local services since 2013 that disproportionally affected deprived areas might have had a substantial impact on health, though we were not able to completely rule out potentially lagged impacts of earlier government funding cuts from 2010 on life expectancy. Increasing investment while prioritising deprived areas could reduce health inequalities.

However, reductions in public spending could plausibly have contributed to declining life expectancy.^12^ The measures introduced by the UK Government to reduce public spending following the 2008 financial crisis disproportionately affected local government, with local authorities facing cuts in central government funding of nearly 50% between 2010 and 2017.^13^ Local government services in England cover a wide range of services including housing, social care, public health, cultural, planning, and environmental services. Consistent evidence supports that these services can have an effect on health, and therefore reducing funding for these services could adversely affect health.^14^, ^15^, ^16^ This reduction in fiscal support has disproportionately affected poorer areas, where the need for services is typically greater.^17^

In addition to overall cuts, the introduction of the Business Rates Retention Scheme in 2013 changed how funds are distributed between local governments in England.^18^ Before 2013, all funds raised through this scheme, the local taxation on business properties, were centrally pooled and redistributed to local government according to regularly updated assessments of need. In 2013, a new policy allowed councils to retain 50% of their business rates as a local share, before the rest is redistributed to local authorities through the Revenue Support Grant (RSG), a grant provided by central government to support local governments' general expenditure on any service. This grant is also allocated on the basis of an assessment of need; however, the assessment was fixed in 2013 and has not been updated since (appendix p 1).^18^ Authorities with increased business rates, for example through economic growth, have benefited from the additional income generated locally. Changes in funding from these sources since 2013 are therefore unlikely to be directly affected by changes in local needs for services, and therefore analysis of the association between funding changes and health outcomes will be less affected by reverse causality, as aforementioned.

Income from RSG and the Business Rates Retention Scheme make up a substantial portion of local government funding (approximately 25% or £26 billion in 2013), and changes in this funding are likely to have had an effect on service provision that could in turn influence life expectancy. Therefore, we aimed to investigate whether local authority areas that showed greater reductions in RSG and business rates income, henceforth referred to as central government funding, between 2013 and 2017 had more adverse trends in life expectancy and premature mortality.

## Methods

### Study design

We did a longitudinal ecological study of 147 of the 152 upper-tier local authorities in England, between 2013 and 2017, using fixed-effects regression. We excluded the City of London and the Isles of Scilly because of their small populations. We also excluded Dorset, Bournemouth, and Poole, given that boundary changes meant that consistent data for these local authorities were not available.

### Data sources and measures

Our primary outcome variable was male and female life expectancy at birth between 2013 and 2017 by local authority in England. Our secondary outcome variables were male and female life expectancy at age 65 years and premature (younger than 75 years) age-standardised mortality from all causes. All measures were provided by Public Health England and are calculated over rolling 3 year intervals to account for annual fluctuations in mortality in relatively small populations. In our analyses of annual trends, we took the middle calendar year as the reference year.

The main exposure variable was annual per-capita allocation of central government funding, defined here as the sum of income from RSG and retained Business Rates to local authorities between the financial years 2013–14 and 2017–18, obtained from the revenue out-turn summary tables, published annually by the UK Ministry of Housing, Communities, and Local Government. The funding calculation methods are presented (appendix p 5). We adjusted all values for inflation to 2017 prices using the gross domestic product deflator,^19^ and we calculated per-capita values using Office for National Statistics (ONS) midyear population estimates.

A potential confounder in this analysis is the trend in overall economic growth in each local authority, because this factor could increase income through retained Business Rates and contribute to increased life expectancy.^20^ To control for these factors in our analysis, we obtained data on unemployment, denoted as a percentage of the economically active population aged 16 years or older, and the average annual Gross Disposable Household Income (GDHI) estimates for each local authority, supplied by the UK ONS. In additional analyses, we allocated local authorities to five equal groups on the basis of their mean income-deprivation domain scores on the 2015 Index of Multiple Deprivation.^21^

### Statistical analysis

We first graphically explored the unadjusted association between changes in life expectancy at birth and changes in central government funding to each local authority, by sex. We then used a set of linear fixed-effects regression models to estimate the association between change in central government funding and change in each of our health outcomes, within each local authority, after adjusting for unemployment rate and average GDHI. The fixed-effects approach removes unobserved confounders that vary between local authorities but are constant over time.^22^ We also included an annual-trend term to adjust for the national long-term trend in health outcomes.

In all models, we used robust clustered SEs to reflect the fact that populations were not sampled independently, and to ensure that SEs were robust to serial correlation in the data. We estimated the models separately using male and female health outcomes (appendix p 6).

We used the predicted marginal effects from these models to estimate trends in life expectancy at birth that would be expected across all of England and in local authorities in the most and least deprived quintiles, if funding for local government had remained the same as in 2013, and graphically compared expected and observed trends. Finally, we used the predicted marginal effects to estimate the additional number of premature deaths during this period compared with the estimated number of premature deaths if funding had been maintained the same as in 2013. We used R 4.0.2 for the statistical analysis.

### Robustness tests

We did several tests to assess the robustness of our findings, including tests for normality, linearity, collinearity, and heteroscedasticity (appendix p 7). To investigate whether results are sensitive to our model specification, we estimated several alternative models, including the following: controlling for annual fixed-effects to account for annual shocks, such as severe flu seasons; log transforming exposure and outcomes to account for potential diminishing returns from investment on life expectancy; and including population as an offset to account for potential spurious relationships caused by mathematical coupling,^23^ because both the outcomes (life expectancy and mortality) and exposure (funds per capita) are derived using population estimates. Because adverse trends in life expectancy could be related to other factors that have disproportionately affected deprived areas, we estimated a model allowing for differing annual trends by quintile of deprivation. Additionally, we estimated a model accounting for differing annual trends by region to account for differential regional trends in access to health care. Given that London has had different trends in life expectancy since the early 2000s, we estimated a model excluding London local authorities, to examine whether our results were influenced by other factors inherent to the region. We also tested for potential confounding effects of internal migration and ethnicity. To explore whether our results are sensitive to our use of life expectancy measures calculated over rolling 3 year periods, we calculated models using single-year data.

Finally, trends in life expectancy after 2013 could possibly be associated with pre-existing trends, originating after the 2010 austerity measures. For this purpose, we did a supplementary difference-in-differences analysis to test whether changes in life expectancy had occurred after the 2013 changes in funding. We identified the top third of local authorities with the largest cuts in central government funding between 2013 and 2017, and compared the change in life-expectancy trends in this group to the rest of the local authorities in England who received less severe cuts. Information on additional data sources used and detailed alternative model results are presented (appendix p 13).

### Role of the funding source

The funders of the study had no role in study design, data collection, data analysis, data interpretation, or writing of the report. All authors had full access to all data in the study. The corresponding author had final responsibility for the decision to submit for publication.

## Results

**Figure 1:**
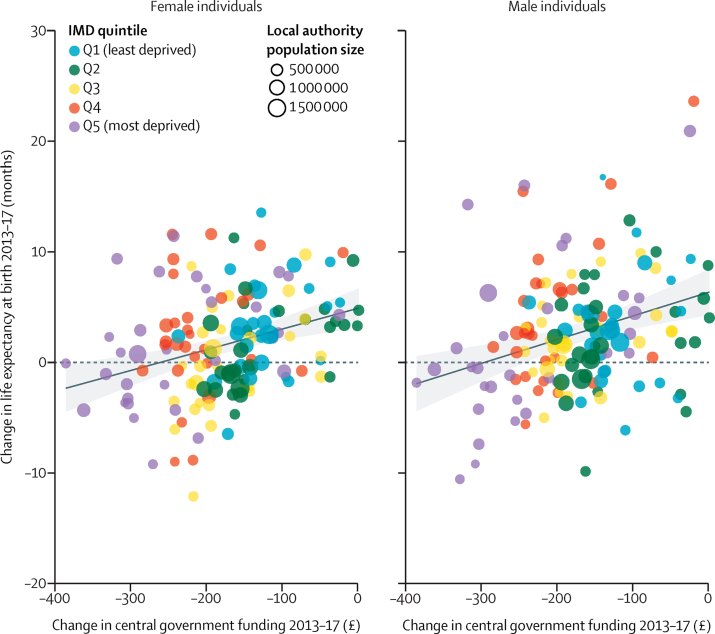
Association between per-person change in central government funds to each local authority area and change in life expectancy at birth for men and women between 2013 and 2017 The solid line represents the linear fit to the data and the shaded area represents 95% CIs. IMD=2015 Index of Multiple Deprivation.

Mean per-capita central government funds to local authorities, from the RSG and Business Rates streams, decreased in real terms by 33% or £168 per person (range –£385 to £1) during the study period, from £514 per person on average in 2013 to £346 in 2017. Male life expectancy at birth increased by only 0·4%, from 79·3 years in 2013 to 79·6 years in 2017, whereas female life expectancy increased by only 0·1%, from 83·1 years to 83·2 years during the same time period. We present the unadjusted associations between change in life expectancy and change in central government funding for each local authority between 2013 and 2017 (figure 1). These findings indicate that local authorities that showed a greater reduction in central government funding over this period had slower growth in life expectancy, or indeed a decline, for both men (*r*=0·31, p=0·00014) and women (*r*=0·31, p=0·0001). The results also show that declines in funding and reduced gains in life expectancy appear to be greater in the more deprived local authorities (figure 1).

We present the results from linear fixed-effects multivariable regression models, outlining the association between trends in government funding for local authorities and each of our outcomes (table). For male individuals, a £100 per-person reduction in central government funding to local government was associated with a reduction of approximately 1·3 months in life expectancy at birth (95% CI 0·7–1·9), 0·8 months in life expectancy at age 65 years (0·3–1·3), and four additional deaths under 75 years per 100 000 people (1·6–6·3). For female individuals, a £100 per-person reduction in central government funding to local government was associated with a reduction of 1·2 months in life expectancy at birth (0·7–1·7), 1·1 months in life expectancy at age 65 years (0·7–1·5), and three additional deaths under 75 years per 100 000 people (1·3–4·6).

**Table:** Change in life expectancy and premature mortality for each £100 per-capita reduction in annual central government funds allocated to local authorities in England 2013–2017

	**Estimate**	**95% CI**	**p value**
**Life expectancy at birth, months**			
Male individuals	−1·28	−1·88 to −0·69	<0·0001
Female individuals	−1·19	−1·73 to −0·65	<0·0001
**Life expectancy at age 65 years, months**			
Male individuals	−0·81	−1·32 to −0·29	0·0021
Female individuals	−1·09	−1·48 to −0·71	<0·0001
**Age-adjusted all-cause mortality for those younger than 75 years, deaths per 100 000**			
Male individuals	3·91	6·27 to 1·55	0·0012
Female individuals	2·94	4·61 to 1·27	0·0005

Model results are based on fixed-effects regression (appendix p 10) for male and female measures, adjusted for trends in household income, unemployment rate, and national annual time trends. p values and 95% CIs are based on robust clustered SEs.

**Figure 2:**
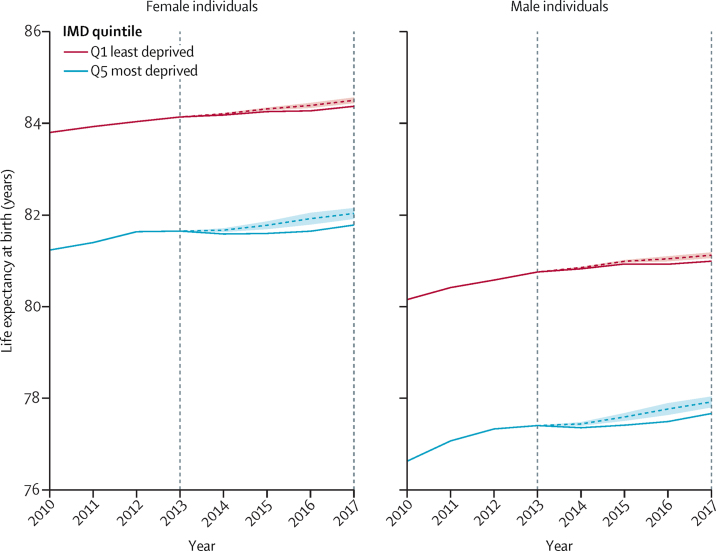
Actual trend in life expectancy at birth by IMD quintile between 2010 and 2017 and expected trend in the absence of cuts to central government funding for local government between 2013 and 2017 Dashed coloured lines show expected trend. Shaded area indicates the 95% CIs of the prediction. Vertical dashed lines show the study period. IMD=2015 Index of Multiple Deprivation.

Overall, in the absence of the cuts over this 5 year period, we estimate that in 2017, male life expectancy at birth could have been 2·2 months higher (1·2–3·2) and female life expectancy could have been 2·0 months higher (1·1–2·9) than observed. We present the trend in life expectancy predicted from the regression models in the most-deprived and least-deprived quintiles of local authorities if funding had remained the same as in 2013, compared with the actual trend (figure 2). As reductions in local government funding were greater in more deprived areas than in less deprived areas, the estimated effect of loss of funding on life expectancy is greater in deprived areas. Over the 5 year period, the most deprived 20% of areas showed a mean decrease in central government funding per capita of £233, compared with a £135 decrease within the 20% least deprived areas. Our model suggests that within the most-deprived quintile of areas, in the absence of cuts, male life expectancy at birth in 2017 could have been 3·0 months higher (95% CI 1·6–4·4) and female life expectancy 2·8 months higher (1·5–4·0). Within the least-deprived quintile of areas, in the absence of cuts, male life expectancy at birth in 2017 could have been 1·7 months higher (0·9–2·5), and female life expectancy at birth in 2017 could have been 1·6 months higher (0·9–2·3). Because of these differential effects by amount of deprivation, we estimate that the cuts in funding could have increased the gap in life expectancy between the most and least deprived quintiles by 3% for men and 4% for women. Using the models for premature mortality, we estimated that overall, the reduction in funds for local government was associated with an estimated additional 9600 deaths in people younger than 75 years (95% CI 3800–15 400) over this 5 year period.

We found that the estimations of the alternative-model specifications, as outlined by the robustness tests, did not markedly change the findings (appendix p 14). Results from the analysis using single-year life-expectancy measures showed no significant change in strength or direction of the association. In difference-in-difference analyses accounting for pre-existing trends in life expectancy from 2010, we found similar effects, with the third of local authorities with the highest cuts showing a greater reduction in life expectancy after 2013 than local authorities receiving less severe cuts (appendix p 11).

## Discussion

Our study suggests that, during a period of large reductions in central government funds for local government in England, areas that had the greatest loss of revenue also showed slower improvements or a decline in life expectancy and premature mortality trends. As funding for the most deprived local authorities decreased to a greater extent than in other local authorities, they showed the most adverse effects, widening health inequalities.

Although this study has not directly investigated causal mechanisms, potential pathways include those affecting mortality in older or vulnerable cohorts via social determinants of health over shorter time frames. Increased mortality could be attributed to decreased spending in adult social care, housing and homelessness prevention, and environmental and regulatory services. Evidence shows that decreased spending on people aged 65 years and older has led to increased use of accident and emergency services.^11^ Social isolation and loneliness are now recognised as important causes of death from cardiovascular disease and stroke,^24^ and decreased spending on social support services might affect mortality through these pathways over short time periods. Decreased investment in housing services has been associated with the sharp rise in homelessness since 2010.^25^ Homelessness is viewed as a key risk factor for drug-related and alcohol-related mortality.^26^, ^27^ In addition, environmental services include a broad range of services targeted at population health, including infectious-disease control, food-and-water safety, and housing standards, which affect conditions associated with higher mortality from cardiovascular, respiratory, and communicable diseases, among others.^28^

Stalling life expectancy in the past decade has been a major public health concern, not only in the UK but also in other European countries. Mortality trends and austerity measures occurred in parallel following the global financial crisis of 2008, and austerity policies have been associated with increased health and social crisis in Europe.^29^ Similar mortality trends have also been observed in the USA, with similar timings but different underlying characteristics, these characteristics being heavily influenced by midlife deaths related to drug and alcohol abuse.^30^ There is some American evidence on the effect of local services on mortality,^31^, ^32^ but the relevance of these studies in international contexts is uncertain because of the underlying differences in health systems and local service delivery.

Several strengths of our analysis enhance its validity. We assessed the natural policy experiment of changing funding for local areas in England using high-quality longitudinal data. By using fixed-effects panel-regression techniques, we were able to control for potentially unobserved time-invariant confounders between local authorities, and controlled for observed differences in economic trends. Our analysis of subnational variation in policy exposure is more informative than a simple analysis of national time trends, given that we can also account for time-varying unobserved confounders that have a similar effect across the country as a whole, such as particularly cold winters. We were able to address endogeneity concerns by identifying local government-funding streams, whereby the change in resources from these streams would not be influenced by changes in needs during the study period.

Some limitations remain, however. There are weaknesses inherent to ecological studies, such as the use of aggregated data and the risk of ecological fallacy. We also cannot rule out the possibility that the associations observed were caused by other confounding factors for which we were not able to control. Other policies introduced or rolled out during this period might also have affected mortality, for example, welfare reforms.^33^, ^34^ However, exposure to changes to the welfare system should still broadly reflect levels of deprivation. When we controlled for differential trends across levels of deprivation, our results were largely unchanged, suggesting that this factor was not a major concern. Nevertheless, unrecognised but temporally coincident causes for the observed trends in life expectancy might be present. Alternative explanations could include, for example, migration. Although we did test the influence of net internal migration on our estimates and found no substantive difference, we could not account for differences in demographic or socioeconomic composition of migrant populations.

Analyses regarding the effects of cuts before 2013 were not possible because of the change in funding policy in 2013. Our main results could be influenced by pre-existing trends before 2013; but, we contend that our supplementary difference-in-difference analysis shows a clear change in trend occurring after 2013 that disproportionately affected local authorities that had the greatest cuts. However, due to the time lagged nature of effects, we cannot rule out the possibility that any impacts of cuts made to government spending in 2010 on life expectancy might not be seen until 2013 or later. Because of the short time series of the study, we were also not able to investigate lagged effects. Expanding the dataset when additional years of data become available would allow a more reliable exploration of lag structures. Finally, we were not able to establish whether the association between funding reductions and reduced life expectancy was caused by disinvestment in specific services. Analysis of specific service budget lines is complicated by the fact that they are not independent; increased spending in one service area usually means reduced spending in another area.

Our results have important implications for policy. They suggest that both the amount and distribution of investment in local government potentially have an effect on health and health inequalities. Policies that are likely to shift resources away from more deprived areas, for example the UK Government's Fair Funding Review^18^ and the proposed expansion of the Business Rate Retention Scheme,^35^ could potentially increase inequalities.

The UK Government has declared that austerity is over and has committed to investing more to support places that have previously been left behind. This commitment has become increasingly important following the global COVID-19 pandemic, which could have disproportionately affected the health and economy of the most disadvantaged places. Fair and equitable investment in local government could potentially redress these inequalities, enabling the country to build back better.

For the **place-based longitudinal data resource** see https://pldr.org/

## Data sharing

All source data used in this study are publicly available. Compiled datasets on central government funding, including the underlying methodology, are openly available to access at the data repository maintained by the authors (place-based longitudinal data resource), or by contacting a.alexiou@liverpool.ac.uk.

## Declaration of interests

We declare no competing interests.
