# Supplementary material for: Local government funding and life expectancy in England: a longitudinal ecological study
Source: Lancet Public Health. 2021 Jul 12;6(9):e641–7. doi: 10.1016/S2468-2667(21)00110-9 (PMC8390384; doi:10.1016/S2468-2667(21)00110-9)
Supplement: Supplementary appendix [file mmc1.pdf]

THE LANCET
Public Health

Supplementary appendix

This appendix formed part of the original submission and has been peer reviewed. We post it as supplied by the authors.

Supplement to: Alexiou A, Fahy K, Mason K, et al. Local government funding and life expectancy in England: a longitudinal ecological study. *Lancet Public Health* 2021; published online July 12. http://dx.doi.org/10.1016/S2468-2667(21)00110-9.

Appendix

Local government financing in England

Local authorities (councils) in England receive income from a combination of domestic (Council Tax) and non-domestic (Business Rates) taxes, central government grants, and sales, fees and charges that they receive for delivering services. They use the majority of this revenue on three types of spending: capital projects (e.g. roads, schools), council housing and local services.[1]

Central government supports local councils' general expenditure through the Business Rates and Revenue Support Grant (RSG), which amount to a quarter of the LAs' total service expenditure. The RSG is a central government grant given to local authorities which can be used to finance revenue expenditure on any service. Business Rates (the amount a business pays in taxes relative to its property value), were pooled nationally and redistributed to local authorities according to a finance settlement that considered their level of need.

Table 1. An outline of the policy intervention. Guidelines broadly adopted from Campbell et al.[2]

Policy	Description
Business Rate Retention Scheme	National policy change regarding local government funding.
Where	Local Authorities in England.
When	Implemented in 2013 and is still in effect.
What and how	The policy introduced substantial changes to the local government funding mechanism by diminishing the redistribution element of business tax income. Previously, all resources where pooled together nationally and distributed according to need. After the policy implementation, only 50% of the resources would be redistributed and the rest would be kept by local authorities where the rateable business is located.
Why	The stated intention of this system is to encourage LAs to grow their local economies, with the incentive of additional revenue.
Variation	Some councils entered a pilot scheme of 100% retention rate, i.e., with no redistribution element in 2017 and 2018. Other small variations occurred, but none took into account local need for services.

In 2013, the government implemented a fundamental change in the way it allocated Business Rates to councils with the introduction of the Business Rate Retention scheme (see table 1 for a brief policy outline).[3] While previously, all the funds received through Business Rates were redistributed to councils, from 2013 onwards 50% of Business Rates were not re-distributed and essentially "retained" by local government (the "local share"). The other 50% was, and is at the time of writing, added and allocated through the RSG (the "central share"). The stated intention of this system is to encourage LAs to grow their local economies, with the incentive of additional revenue. If Business Rate income increased – e.g. due to increases in the number and type of rateable businesses, councils kept 50% of that extra income. Likewise, if business rate income reduced from baseline, they would have lost income. The eventual intention is to implement 100% retention rates[4] while government fiscal support directly though the RSG to be phased out (figure 1).

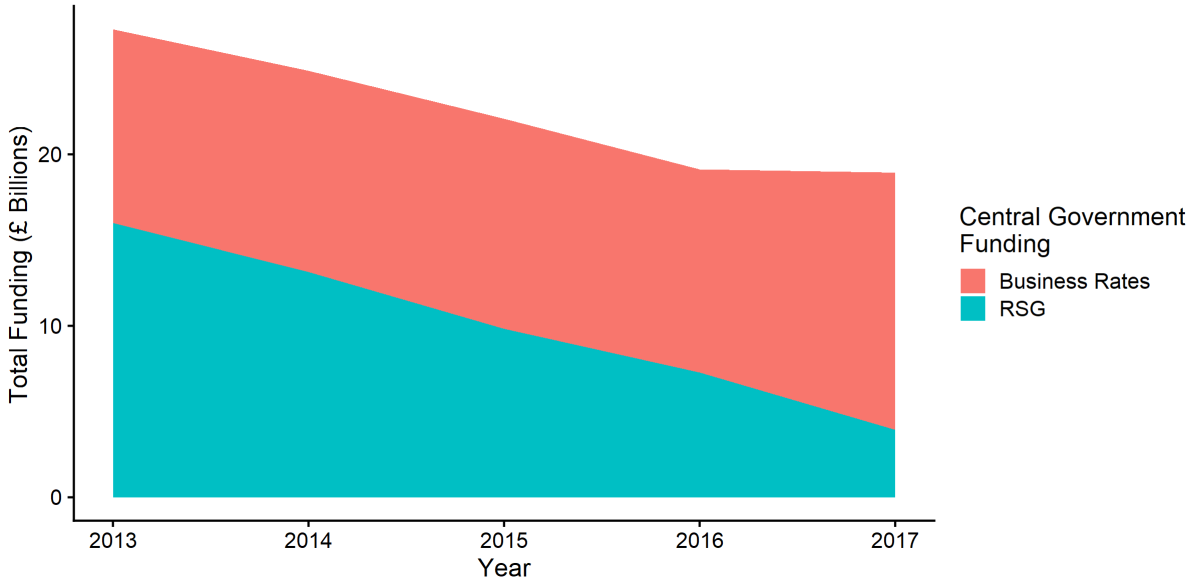

Figure 1. Trends in total central government funding to local government in England from the RSG and Business Rates funding streams between 2013 to 2017. Values have been adjusted using the GDP deflator.

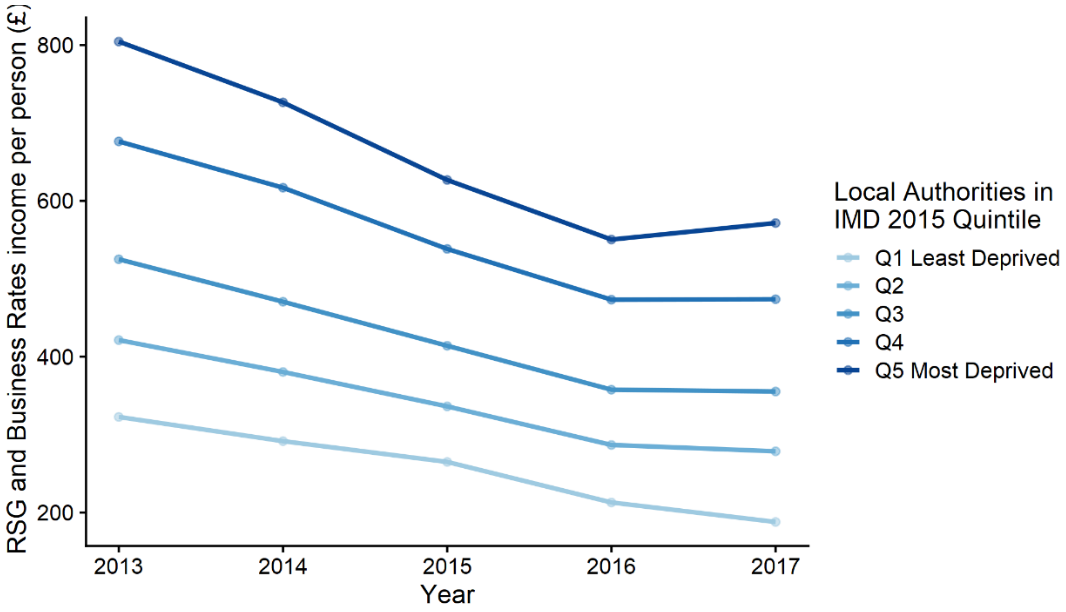

Figure 2. Trends in local government income per person from the RSG and Business Rates between 2013 to 2017 by Local Authority IMD 2015 quintile. Values have been adjusted using the GDP deflator.

The total amount of funds available by the government is determined through a spending review. This amount, along with the estimated funds raised through the retained portion of Business Rates, sets the total amount of funding to be redistributed through the RSG.

How the total funds from the RSG are distributed between individual councils however depends on the "start-up funding assessment". This was set in 2013 using a series of formulae that account for the relative needs of each LA, adjusted by the revenue each council is expected to raise from Council Tax. The start-up funding assessment has not been revised between 2013 and 2017, essentially holding the relative needs between councils constant.

In more detail, starting in 2013, the funding that each local authority received from these sources was scaled so that it was equal to the start-up funding assessment. The baseline figure was adjusted each following year for

every local authority using the share of RSG funding it received in the previous year applied to the total RSG funding agreed in the settlement in the current year, after adjusting for inflation and changes in the business property evaluation. Over this 5-year period, a series of adjustments were also introduced to compensate some LAs that would have been severely affected by changes to the RSG or Business Rate income (top-ups).

In 2016 for example, the government brought in several changes including modifying how RSG takes into account councils' ability to raise income through Council Tax, introducing a transitional grant to compensate some councils most affected by the change, removing tariffs and top-ups for some councils and providing additional funding to rural areas. In 2017, Business Rates increased as a result of a revaluation of business properties.[5] Further changes were introduced in 2017, with 27 councils becoming 100% Business Rate Retention pilots. These councils retained 100% their adjusted Business Rates income and received no RSG. The pilot was set up in such a way that these pilot councils received higher levels of funding than they would have under the 50% Business Rate Retention scheme,[4] and essentially targeting relatively deprived, urban areas, which significantly benefited from the scheme (figure 2).

Whilst the introduction of the scheme in 2013 suggests greater local control of resources, it is still essentially a process for centrally allocating resources to local areas. Business Rates remain a national tax in England that is set by central government. These changes meant that between 2013 and 2017 there was significant variation in total funding, with variation between councils depending on a) the overall funding agreed in the settlement b) their income from retained Business Rates (which in turn depend on business growth and taxation) and c) administrative changes and top-ups as outlined above. As aforementioned, none of these adjustments took into account changes in local need, which was set at baseline.

Aside from the retained Business Rates and RSG, the other two main sources of funding include various Special and Specific Grants and Council Tax. We have not included special and specific grants in our analysis, as these include "targeted" grants for specific purposes, such as the Dedicated Schools Grant, intended to finance e.g. schools, and financed by the Department for Education. The basis of the distribution varies from grant to grant,[3] but their levels are generally determined by national priorities and specific needs of LAs (e.g. education, policing, social care etc.) and increases in these needs will both increase grant income from these sources as well as increase mortality risk (see logic model in figure 2).

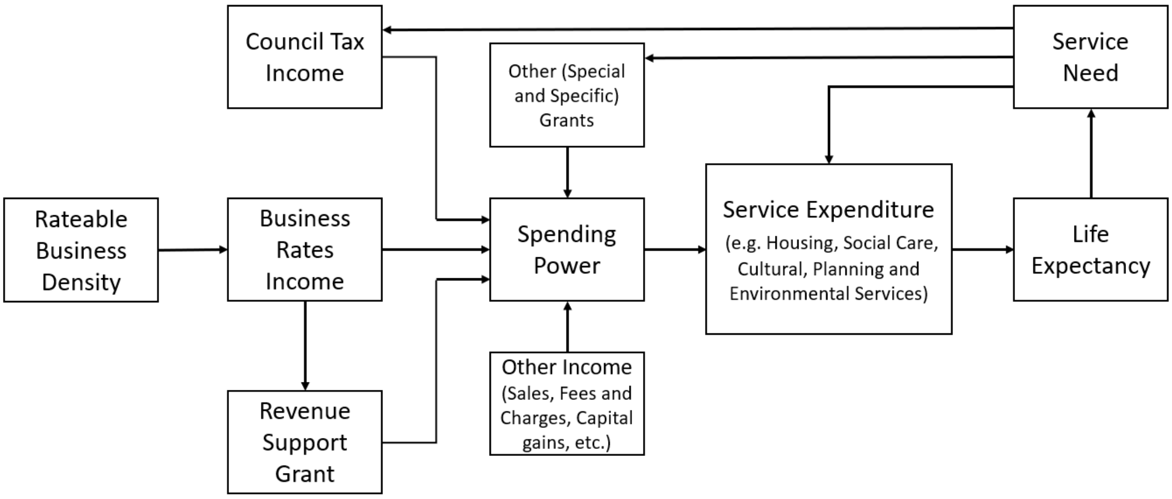

Figure 3. Logic model showing the putative relationship between service expenditure, LA funding and life expectancy.

The last major revenue stream comes through Council Tax (taxation on domestic properties). While Council Tax makes up a large part of LAs' revenue, in 2012 a cap was introduced, restricting the amount that local taxes can be raised each year.[6] Total revenue is largely linked to housing stock, and there is little differentiation from year to year. Any small variation in Council Tax income will also be affected by changes in needs, as groups of people with particular needs - e.g. individuals with disabilities - are exempt from paying Council Tax.

In this framework, by limiting our central government funding measure to income from the RSG and retained Business Rates between 2013, when the new allocation policy was introduced, and 2017, when the latest data are available, we ensured that change within this income stream was no longer directly linked to changing needs within LA populations. Over this time period, analyses using this measure should be less susceptible to biases related to reverse causation.

We also investigated whether effects from cuts in local services started in 2010, when austerity measures were first introduced, and then carried on during our study period. Based on the available evidence, local authorities did try initially to offset to impact of austerity introduced in 2010 by trying to make savings through efficiencies rather than by reducing services,[7] namely through service transformation, excess staffing costs, and redundancy payments. As a response, local councils tried to cut unnecessary costs and (successfully) built-up reserves in the face of the incoming financial uncertainty[8]. In this context we have concluded that cuts in funding were unlikely to have materialized in significant reductions in services until after 2013.

Calculation of LA–level revenue data

Data on central government funding to individual LAs were compiled using the General Fund Revenue Account Outturn - Revenue Outturn Summary (RS) tables, published annually by the UK Department for Communities and Local Government; specifically from columns "Revenue Support Grant" and "Retained Income from Rate Retention Scheme".

To provide a consistent time series of allocations of the RSG and Business Rates income across LAs, figures from all the different types of LAs were mapped to upper tier LAs. Local government structure in England is hierarchical, with two main tiers of local government, upper and lower, that are geographically nested. In addition, there are several other types of LAs, such as police, fire and combined authorities, each with its own area and domain of activity. The allocation of funds within these is similarly reported at multiple levels.

In order to make comparisons possible, all figures from lower tier authorities (i.e. Shire Districts) were aggregated to upper tier level, in particular County Councils, Metropolitan Districts, Unitary Authorities and London Boroughs, using look-up tables provided by the Office for National Statistics (ONS). Where local government organisations spanned more than one upper-tier LA, we apportioned the total allocation to each LA based on their annual population estimates, as provided by ONS. In particular, these LA types are:

- The Greater London Authority
- Combined Authorities
- Police and Crime Commissioner and Chief Constable Authorities
- Fire and Rescue Authorities
- Waste Authorities
- Transport Authorities

All income figures for the above types of authorities have been included in our calculations and compiled into annual figures, with the exception of Park Authorities; however, these are a small number of authorities with relatively low levels of funding and spending. Figures are given on the basis of financial years, i.e. from April 1st to March 31st, and for the analysis of annual trends, the first calendar year was used as reference. The compiled dataset can be found at https://pldr.org/ dataset/29dqv/.

Statistical analysis and regression models

1. Summary description

Our panel data consists of n = 147 areas for T = 5 years. It is a balanced panel with N = 735 observations and no missing data. Statistical analysis was carried out in R 4.0.2. Models were calculated using the "plm" library.

2. Model Formulae

Life expectancy at birth model equation:

$$(1)\quad Life\ Expectancy\ Birth_{i,t} = \beta_1 CGF_{i,t} + \beta_2 Unemployment_{i,t} + \beta_3 GDHI_{i,t} + t + \mu_i + \varepsilon_{i,t}$$

Where *Life Expectancy Birth* is the average life expectancy at birth for males or females, *CGF* is the central government funding, *Unemployment* is the unemployment rate and *GDHI* is the average gross disposable household income for each *i* English LA area and *t* year. A dummy variable indicating each LA is denoted by μ, *t* is an annual time-trend and ε denotes the time-varying error term.

Life expectancy at 65 years of age model equation:

$$(2)\quad Life\ Expectancy\ 65_{i,t} = \beta_1 CGF_{i,t} + \beta_2 Unemployment_{i,t} + \beta_3 GDHI_{i,t} + t + \mu_i + \varepsilon_{i,t}$$

Where *Life Expectancy 65* is the average life expectancy at 65 years of age for males or females, *CGF* is the central government funding, *Unemployment* is the unemployment rate and *GDHI* is the average gross disposable household income for each *i* English LA area and *t* year. A dummy variable indicating each LA is denoted by μ, *t* is an annual time-trend and ε denotes the time-varying error term.

Premature mortality model equation:

$$(3)\quad Premature\ Mortality_{i,t} = \beta_1 CGF_{i,t} + \beta_2 Unemployment_{i,t} + \beta_3 GDHI_{i,t} + t + \mu_i + \varepsilon_{i,t}$$

Where P*remature Mortality* is the age-standardised all-cause mortality rate under the age of 75 for males or females, *CGF* is the central government funding, *Unemployment* is the unemployment rate and *GDHI* is the average gross disposable household income for each *i* English LA area and *t* year. A dummy variable indicating each LA is denoted by μ, *t* is an annual time-trend and ε denotes the time-varying error term.

3. Regression Diagnostics

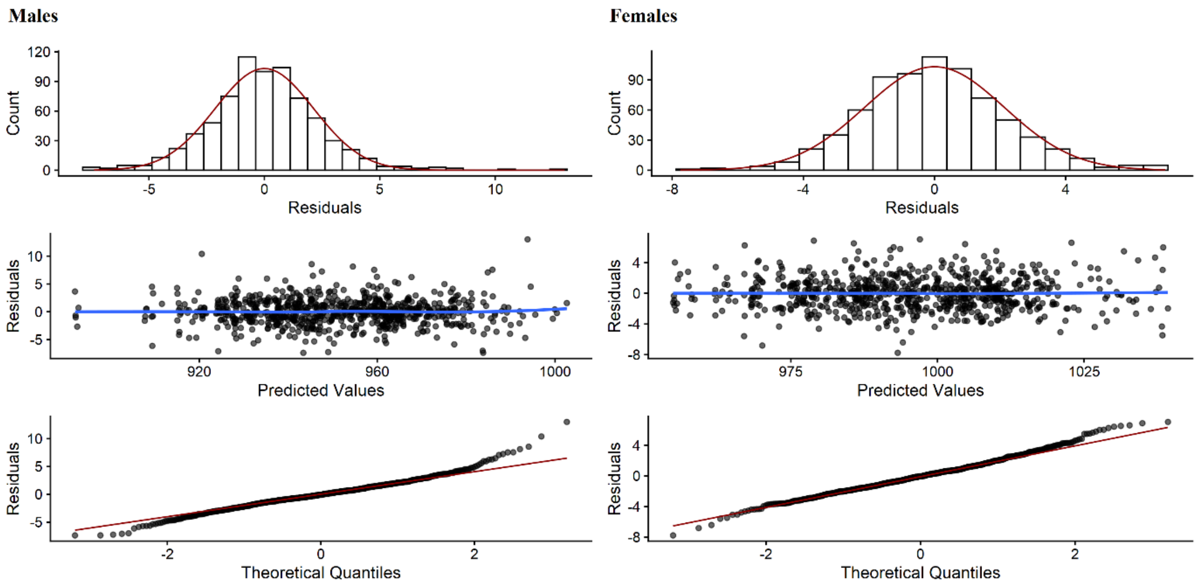

Figure 4. Regression residual distribution (histogram), predicted vs. residual values with Local Polynomial Regression Fitting (LOESS) and quantiles of residuals against theoretical quantiles of normal distribution (Q-Q plot) for model equation (1), for male and female life expectancy at birth as noted.

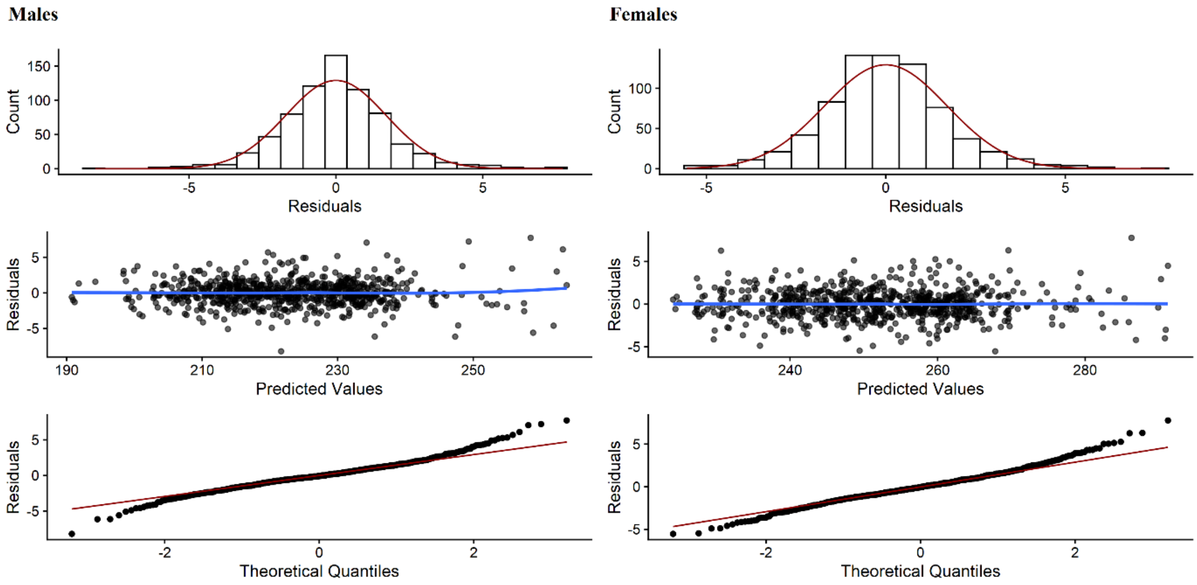

Figure 5. Regression residual distribution (histogram), predicted vs. residual values with Local Polynomial Regression Fitting (LOESS) and quantiles of residuals against theoretical quantiles of normal distribution (Q-Q plot) for model equation (2), for male and female life expectancy at 65 years of age as noted.

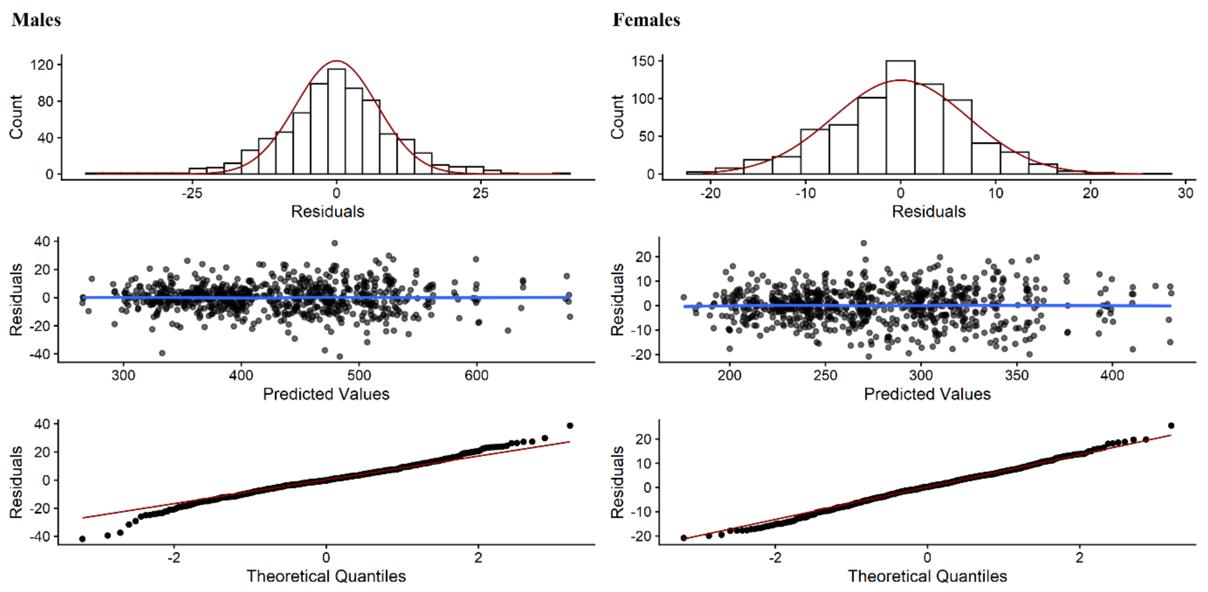

Figure 6. Regression residual distribution (histogram), predicted vs. residual values with Local Polynomial Regression Fitting (LOESS) and quantiles of residuals against theoretical quantiles of normal distribution (Q-Q plot) for model equation (3), for male and female age-standardised all-cause mortality rate under the age of 75 as noted.

Table 2. Variable Inflation Factors of the independent variables for model equations (1) – (3).

Variance Inflation Factors	
Central Government Funding	3·802
Unemployment Rate	2·232
Gross Disposable Household Income	1·659
Year	4·343

Table 3. Variance-Covariance Matrices of the main parameters for model equations (1) – (3), for males and females as noted.

Male life expectancy at birth

	Central Government Funding	Unemployment Rate	GDHI	Year
Central Government Funding	0·060	-0·006	0·006	0·022
Unemployment Rate	-0·006	0·009	0·0004	0·004
GDHI	0·006	0·0004	0·045	-0·009
Year	0·022	0·004	-0·009	0·021

Female life expectancy at birth

	Central Government Funding	Unemployment Rate	GDHI	Year
Central Government Funding	0·049	-0·005	0·004	0·018
Unemployment Rate	-0·005	0·007	0·0003	0·003
GDHI	0·004	0·0003	0·036	-0·007

Year	0·018	0·003	-0·007	0·017

Male life expectancy at 65 years of age

	Central Government Funding	Unemployment Rate	GDHI	Year
Central Government Funding	0·034	-0·003	0·003	0·012
Unemployment Rate	-0·003	0·005	0·0002	0·002
GDHI	0·003	0·0002	0·025	-0·005
Year	0·012	0·002	-0·005	0·012

Female life expectancy at 65 years of age

	Central Government Funding	Unemployment Rate	GDHI	Year
Central Government Funding	0·031	-0·003	0·003	0·011
Unemployment Rate	-0·003	0·004	0·0002	0·002
GDHI	0·003	0·0002	0·023	-0·005
Year	0·011	0·002	-0·005	0·011

Premature mortality rate, males

	Central Government Funding	Unemployment Rate	GDHI	Year
Central Government Funding	1·023	0·095	-0·094	-0·376
Unemployment Rate	0·095	0·148	0·007	0·073
GDHI	-0·094	0·007	0·755	-0·151
Year	-0·376	0·073	-0·151	0·356

Premature mortality rate, females

	Central Government Funding	Unemployment Rate	GDHI	Year
Central Government Funding	0·534	0·050	-0·049	-0·197
Unemployment Rate	0·050	0·077	0·003	0·038
GDHI	-0·049	0·003	0·395	-0·079
Year	-0·197	0·038	-0·079	0·186

4. Regression analysis results

Table 4 shows the regression analysis results for equations (1) – (3) for males and females as noted. Models annotated as (1) and (2) show the change in life expectancy for each increase in central government funding by £100 per capita. Models annotated as (3) show the change in premature mortality rate for each decrease in central government funding by £100 per capita. Table 5 shows similar results for the unadjusted association of central government funding to primary outcomes without controlling for the effects of unemployment rate and GDHI.

Table 4. Fixed effects regression results for the main models.

Model Results

	Primary outcome					
	Males – Life expectancy at birth (months)	Females – Life expectancy at birth (months)	Males – Life expectancy at 65 (months)	Females - Life expectancy at 65 (months)	Males – Premature mortality rate (per 100,000)	Females – Premature mortality rate (per 100,000)
**Independent variables**	(1)	(1)	(2)	(2)	(3)	(3)
Central Government Funding (£100s)	1·284***	1·193***	0·806***	1·094***	3·908***	2·943***
	(0·304)	(0·276)	(0·262)	(0·198)	(1·203)	(0·853)
Unemployment Rate (%)	-0·019	-0·046	0·016	0·006	0·336	0·151
	(0·121)	(0·086)	(0·102)	(0·084)	(0·467)	(0·295)
Average GDHI (£1000s)	0·986***	0·804***	0·604***	0·449***	-2·676**	-2·285***
	(0·204)	(0·175)	(0·177)	(0·164)	(1·105)	(0·735)
Year	0·970***	0·695***	0·951***	0·712***	-3·226***	-1·825***
	(0·188)	(0·150)	(0·127)	(0·130)	(0·662)	(0·439)
Observations	735	735	735	735	735	735
(within) R^2	0·184	0·123	0·277	0·129	0·135	0·092
Adjusted R^2	-0·025	-0·102	0·092	-0·094	-0·087	-0·142
F Statistic (df = 4; 584)	32·948***	20·468***	56·050***	21·658***	22·781***	14·743***

Note: *p<0·05; **p<0·01; ***p<0·001
Robust clustered standard errors are reported in parenthesis.
Premature mortality rate refers to age-standardised, all-cause, under 75 years old mortality rate.

Table 5. Fixed effects regression results for the main models, unadjusted for unemployment rate and GDHI.

Unadjusted Model Results

	Primary Outcome					
	Males – Life expectancy at birth (months)	Females – Life expectancy at birth (months)	Males – Life expectancy at 65 (months)	Females - Life expectancy at 65 (months)	Males – Premature mortality rate (per 100,000)	Females – Premature mortality rate (per 100,000)
Central Government Funding (£100s)	1·144***	1·058***	0·738***	1·040***	3·341***	2·547***
	(0·317)	(0·258)	(0·245)	(0·185)	(1·200)	(0·839)
Year	1·181***	0·882***	1·066***	0·801***	-3·939***	-2·366***
	(0·190)	(0·144)	(0·131)	(0·094)	(0·668)	(0·397)
Observations	735	735	735	735	735	735
(within) R^2	0·153	0·095	0·259	0·116	0·120	0·071
F Statistic (df = 2; 586)	53·118***	30·841***	102·559***	38·448***	39·818***	22·239***

Note: *p<0·1; **p<0·05; ***p<0·01
Robust clustered standard errors are reported in parenthesis.
Premature mortality rate refers to age-standardised, all-cause, under 75 years old mortality rate.

Difference in Difference analysis showing the effects of 2013-2017 cuts in funding on change from pre-existing trends in life expectancy.

1. Methods

In this section we provide supplementary difference-in-differences[10] analysis investigating how trends in life expectancy changed after 2013 in areas experiencing greater cuts in their Central Government Funding after 2013 compared to areas that had experienced less severe cuts. This allows us to account for pre-existing trends originating after the 2010 austerity measures.

Firstly, we identify a group of local authorities that have experienced relatively high cuts in central government funding (2013-2017), defined as the third of local authorities with the greatest cut. We then compare the trends in life expectancy before (2010-2013) and after (2013-2017) in these two groups. Initially, we plot this trend as the change from the baseline year (2010). We then test whether the trends prior to 2013 were approximately parallel between the two groups by fitting a regression model on the 2010-2013 data of the form:

$$(4)\quad Life\ Expectancy\ Birth_{i,t} = \beta_1 Group_{i,t} + \beta_2 Year_t + \beta_3 Year * Group_{i,t} + \varepsilon_{i,t}$$

Where *Life Expectancy Birth* is the average life expectancy at birth for males or females, *Group* is a dummy variable indicating whether they are in high-cuts group or not, and *Year* is an annual time-trend. The interaction term ($Year * Group$) indicates whether there was a significant difference in trends between these groups before 2013.

Finally, we estimate the change (difference) in life expectancy in the high-cuts group relative to the change in the comparison group, i.e., the difference in differences. This is done by estimating the following regression on our data:

$$(5)\quad Life\ Expectancy\ Birth_{i,t} = \beta_1 Group_{i,t} + \beta_2 Year_t + \beta_3 After_t + \beta_4 After * Group_{i,t} + \varepsilon_{i,t}$$

Where '*After'* is a dummy variable indicating the time period 2013-2017 or zero otherwise. β_4 is the difference-in-difference parameter indicating the change in the high-cuts group relative to the change in the group of LAs with less severe cuts. Generalised Estimating Equations were used to account for the longitudinal nature of the data.

2. Results

Figure 7 below graphically describes the trend in life expectancy relative to the baseline in each group. We can clearly see a reduction in life expectancy after 2013 that is greatest in the high-cuts group, indicating a divergence from the pre-existing trend in the high-cuts group.

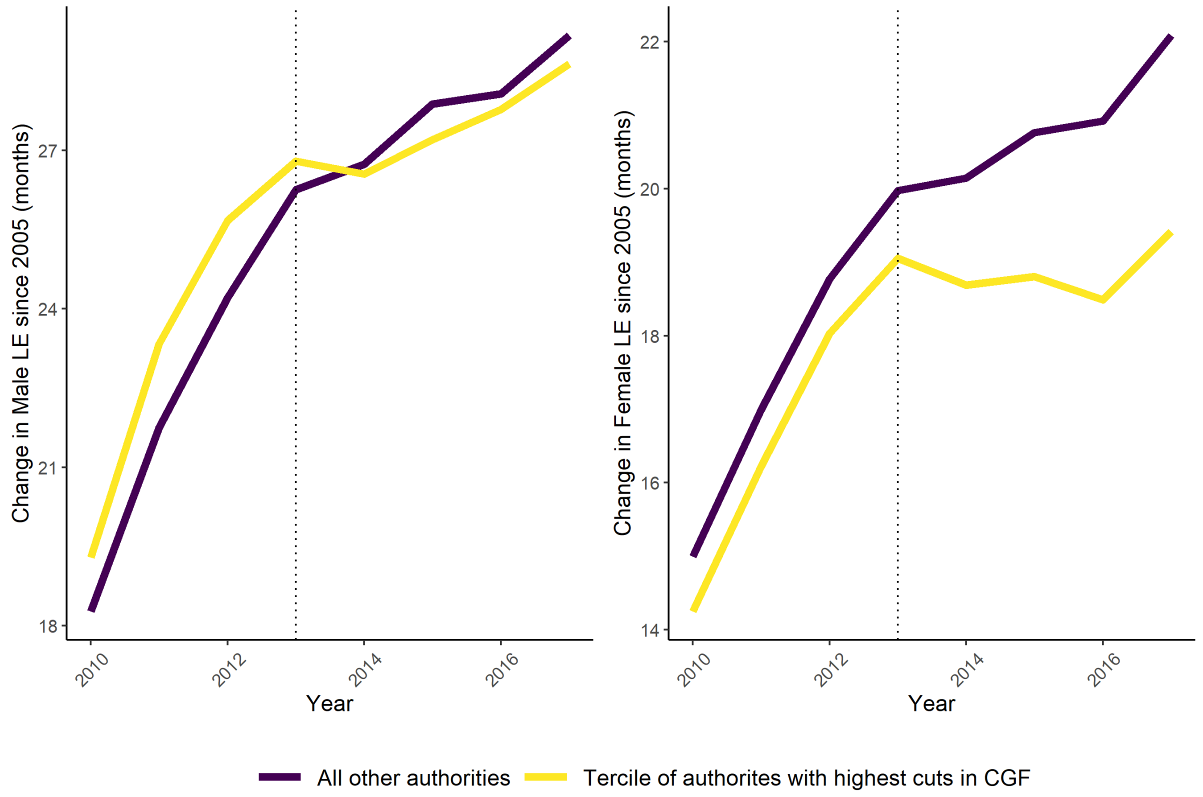

Figure 7. Association of life expectancy at birth gains since 2010 and level of cuts in central government funding between the years 2013-2017. The yellow line represents LAs who experienced the greatest funding cuts between 2013 to 2017.

We then tested whether trends were parallel in both groups before 2013 and find that there was no evidence of diverging trends between 2010-2013 for male (p=0·8) and female (p=0·4) life expectancy at birth. In fact, we find no association between the level of cut (2013-2017) and previous trends in life expectancy (2010-2013) at the local authority level (p=0·3), suggesting this is unlikely to be confounder in the primary analysis in our paper.

From the difference-in-difference analysis, we then estimate that the third of LAs receiving the greatest cuts in central funding experienced 2·3 months (95% CI: 0·67 to 4·02, p=0·005) and 1·8 months (95% CI: 0·54 to 3·15, p=0·006) reduction in male and female life expectancy respectively, compared to LAs receiving less severe cuts.

3. Conclusion

The analysis supports our conclusions found in the main paper indicating that cuts from 2013 in Central Government Funding were associated with more adverse trends in life expectancy, and that this was not due to a continuation of pre-existing trends.

Alternative Specifications

1. Data sources, specification and model results for the analysis using single-year life expectancy at birth measures.

The creation of single-year life expectancy at birth measures for males and females involved data on the number of deaths by 5-year age groups, Lower layer Super Output Area in England and Wales, mid-year to mid-year period, between July 1st 2000 and June 30th 2018, as supplied by ONS. We aggregated these at local authority level and used them along ONS mid-year population estimates by local authority, 5-year age group and sex in order to calculate the life expectancy measures. We followed the exact ONS methodology when creating these.

Some limitations include that annual figures are based on a July to June basis, and not calendar years. Since our exposure variable regarded fiscal years (April 1st to March 31st), there is an inherent 3-month lag between annual spending and annual deaths occurred. Furthermore, the dataset only account for deaths that were registered by December 31st 2018. As registrations can take more than 180 days since occurrence, there might be some bias for the last (2017-18) year of data. Lastly, the last age group is for which data on death occurrences is supplied is the 85+ age group. This is different from the ONS methodology which includes a 90+ age group. This might have introduced bias in the calculation of single-year life expectancy measures.

For the model, we used the exact same specification as our main model, although we only present results for our main outcome, life expectancy at birth, for males and females. Model results are based on fixed effects regression as shown in Appendix 3, equation (1), for male and female measures as noted, adjusted for trends in household income, unemployment rate, and national annual time trends. Estimates show the change in life expectancy for each increase in central government funding by £100 per capita. P-values and confidence intervals reported are based on robust clustered standard errors· Results are presented in table 6.

Table 6. Results from model estimation using single-year life expectancy at birth measures.

Model results

	Primary outcome – calculated based on deaths within a single year	
	Males – Life expectancy at birth (months)	Females – Life expectancy at birth (months)
	(1)	(1)
Central Government Funding (£100s)	1·431*	1·898***
	(0·606)	(0·530)
Unemployment Rate (%)	0·435*	0·256
	(0·232)	(0·219)
Average GDHI (£1000s)	0·672	1·229**
	(0·436)	(0·704)
Year	0·767**	0·170
	(0·328)	(0·350)
Observations	735	735
(Within) R^2	0·025	0·048
Adjusted R^2	-0·226	-0·196
F Statistic (df = 4; 584)	3·680***	7·391***

Note: *p<0·05; **p<0·01; ***p<0·001
Robust clustered standard errors are reported in parenthesis

2. Other alternative model specifications

For the alternative models, we used the exact same specification as our main model. Model results are based on fixed effects regression as shown in Appendix 3, equations (1) – (3), for male and female measures as noted, adjusted for trends in household income, unemployment rate, and national annual time trends. Estimates show the change in life expectancy for each increase in central government funding by £100 per capita, except for Models annotated as (3) which show the change in premature mortality rate for each decrease in central government funding by £100 per capita. P-values and confidence intervals reported are based on robust clustered standard errors. Results are presented in table 7.

Regarding additional data sources, data on total annual number of business (local units) where provided by ONS, recording the number of local units that were live at a reference date every March. A local unit is an individual site (for example a factory or shop) also referred to as a workplace.

Data on internal migration were provided by the ONS. The variable used was the net internal migration flows, calculated as the total internal migration inflow to an area minus the internal migration outflow from said area to other areas. Inflows or outflows with origins or destinations respectively located outside England were excluded from the calculations.

Data on ethnicity estimates were provided by the ONS Annual Population Survey. The variable used was the percentage of population who are white UK nationals.

Table 7. Comparisons between the main model and alternative model specifications

Alternative Model Comparisons

	**Outcomes**					
**Central Government Funding coefficient for model:**	Males – Life expectancy at birth (months)<br>(1)	Females – Life expectancy at birth (months)<br>(1)	Males – Life expectancy at 65 (months)<br>(2)	Females - Life expectancy at 65 (months)<br>(2)	Males – Premature mortality rate (per 100,000)<br>(3)	Females – Premature mortality rate (per 100,000)<br>(3)
Main model	1·284***<br>(0·304)	1·193***<br>(0·276)	0·806***<br>(0·262)	1·094***<br>(0·198)	3·908***<br>(1·203)	2·943***<br>(0·853)
Model with differing annual trends by region	0·802***<br>(0·292)	0·764***<br>(0·267)	0·455**<br>(0·257)	0·732***<br>(0·190)	2·696***<br>(1·288)	1·797**<br>(0·888)
Model with fixed annual effects	1·099***<br>(0·391)	0·961**<br>(0·425)	0·575<br>(0·353)	0·848**<br>(0·355)	3·777**<br>(1·492)	2·729***<br>(0·647)
Model excluding LAs within the London region	1·373***<br>(0·266)	1·231***<br>(0·383)	0·882***<br>(0·262)	1·048***<br>(0·260)	3·158**<br>(1·318)	3·342***<br>(1·232)
Model with log-transformed variables	0·006***<br>(0·002)	0·005***<br>(0·001)	0·019***<br>(0·005)	0·020***<br>(0·005)	0·056***<br>(0·013)	0·057***<br>(0·015)
Model with values not normalised by population (log-transformed)	0·007***<br>(0·002)	0·006***<br>(0·001)	0·020***<br>(0·005)	0·020***<br>(0·004)	0·058***<br>(0·013)	0·059***<br>(0·015)
Model with different annual trends in areas by deprivation quintiles	1·614***<br>(0·338)	1·404***<br>(0·320)	1·039***<br>(0·274)	1·286***<br>(0·220)	5·148***<br>(1·310)	3·622***<br>(0·873)
Model additionally controlling for the total number of businesses each year	1·197***<br>(0·298)	1·125***<br>(0·275)	0·752***<br>(0·260)	1·038***<br>(0·197)	3·579***<br>(1·176)	2·797***<br>(0·837)
Model additionally controlling for net internal migration flows	1·278***<br>(0·307)	1·187***<br>(0·276)	0·803**<br>(0·263)	1·093***<br>(0·198)	3·862**<br>(1·200)	2·929***<br>(0·854)
Model additionally controlling for the percentage of population who are white UK nationals	1.271***<br>(0.305)	1.176***<br>(0.271)	0.789***<br>(0.260)	1.074***<br>(0.191)	-3.907***<br>(0.210)	-2.919***<br>(0.854)

Note: *p<0·05; **p<0·01; ***p<0·001
Robust clustered standard errors are reported in parenthesis.
Premature mortality rate refers to age-standardised, all-cause, under 75 years old mortality rate.

References

1 Department for Communities and Local Government. A guide to the local government finance settlement in England. Crown copyright, 2013. https://assets.publishing.service.gov.uk/government/uploads/system/uploads/attachment_data/file/266886/LGFS_Guide.pdf (accessed June 7, 2020).

2 Campbell M, Katikireddi SV, Hoffmann T, Armstrong R, Waters E, Craig P. TIDieR-PHP: a reporting guideline for population health and policy interventions. BMJ 2018; 361: k1079.

3 Sandford M. Reviewing and reforming local government finance. House of Commons Library, Briefing Paper 07538, 2020. https://commonslibrary.parliament.uk/research-briefings/cbp-7538/ (accessed July 6, 2020).

4 Smith NA, Phillips D, Simpson P. 100% business rate retention pilots: what can be learnt and at what cost? IFS, 2018; published online April 12. DOI:10.1920/BN.IFS.2018.BN0233 (accessed July 29, 2020).

5 Valuation Office Agency. Non-domestic rating: Revaluation 2017. GOV.UK. 2017; published online Dec 7. https://www.gov.uk/government/collections/non-domestic-rating-revaluation-2017 (accessed July 29, 2020).

6 Sandford M. Council tax: local referendums. House of Commons Library, Briefing Paper 05682, 2019. https://researchbriefings.files.parliament.uk/documents/SN05682/SN05682.pdf (accessed July 6, 2020).

7 National Audit Office. Financial sustainability of local authorities 2014. Ministry of Housing, Communities & Local Government, 2014. https://www.nao.org.uk/wp-content/uploads/2014/11/Financial-sustainability-of-local-authorities-20141.pdf (accessed July 22, 2020).

8 National Audit Office. The impact of funding reductions on local authorities. Ministry of Housing, Communities & Local Government, 2014. https://www.nao.org.uk/wp-content/uploads/2014/11/Impact-of-funding-reductions-on-local-authorities.pdf (accessed July 22, 2020).

9 Office for National Statistics. Guide to calculating national life tables. https://www.ons.gov.uk/peoplepopulationandcommunity/healthandsocialcare/healthandlifeexpectancies/methodologies/guidetocalculatingnationallifetables (accessed Jan 27, 2021).

10 Wooldridge JM. Econometric Analysis of Cross Section and Panel Data, second edition edition. Cambridge, Mass: MIT Press, 2010.
